# Case studies of innovative medical device companies from India: barriers and enablers to development

**DOI:** 10.1186/1472-6963-13-199

**Published:** 2013-05-30

**Authors:** Szymon Jarosławski, Gayatri Saberwal

**Affiliations:** 1 Institute of Bioinformatics and Applied Biotechnology, Biotech Park, Electronics City Phase I, Bangalore, India

## Abstract

**Background:**

Over 75% of the medical devices used in India are imported. Often, they are costly and maladapted to low-resource settings. We have prepared case studies of six firms in Bangalore that could contribute to solving this problem. They have developed (or are developing) innovative health care products and therefore are pioneers in the Indian health care sector, better known for its reverse engineering skills. We have sought to understand what enablers and barriers they encountered.

**Methods:**

Information for the case studies was collected through semi-structured interviews. Initially, over 40 stakeholders of the diagnostics sector in India were interviewed to understand the sector. However the focus here is on the six featured companies. Further information was obtained from company material and other published resources.

**Results:**

In all cases, product innovation has been enabled by close interaction with local medical practitioners, links to global science and technology and global regulatory requirements. The major challenges were the lack of guidance on product specifications from the national regulatory agency, paucity of institutionalized health care payers and lack of transparency and formalized Health Technology Assessment in coverage decision-making. The absence of national evidence-based guidelines and of compulsory continuous education for medical practitioners were key obstacles in accessing the poorly regulated and fragmented private market.

**Conclusions:**

Innovative Indian companies would benefit from a strengthened capacity and interdisciplinary work culture of the national device regulatory body, institutionalized health care payers and medical councils and associations. Continuous medical education and national medical guidelines for medical practitioners would facilitate market access for innovative products.

## Background

In the case of drugs, due to its strong reverse engineering skills, India is virtually self-sufficient. In contrast, 75% of the annual purchase of devices and diagnostics comes from imports [1]. A WHO report on medical devices pointed out that: “almost all devices present in developing countries have been designed for use in industrialized countries” [2]. Consequently, they are often unaffordable and are maladapted to low resource settings.

Whereas rural health care providers are a documented source of grassroots technical innovation on a micro scale [3] the private industry world-wide has valuable expertise in the development of medical devices for mass use [2]. However, the industry has traditionally perceived that developing-world markets are too small to justify the development of new products [2,4]. Thus, over the past decade, a number of push and pull incentives have been proposed by international public health organizations, non-governmental organizations (NGOs) and donors in order to incentivize the western industry to undertake research and development (R&D) addressing the specific needs of the developing world [4,5], although market access challenges of this industry in such markets have been well-documented [6]. More recent is health technology innovation, largely by young companies located in developing countries, where the companies perceive local markets as the main focus of their R&D strategy [7,8].

Important to health technology innovation is Health Technology Assessment (HTA), defined as the “systematic evaluation of the properties and effects of a health technology, addressing the direct and intended effects of this technology, as well as its indirect and unintended consequences, and aimed mainly at informing decision making regarding health technologies” (http://htaglossary.net). In industrialized countries, there is a growing interest in interactions among bodies concerned with HTA, coverage (institutional purchasing or reimbursement), and regulation with whom the industry needs to engage in order to develop novel products that can reach patients [9]. Improving such interactions is believed “to speed patient access to valuable products” and “to remove unnecessary barriers to successful development and appropriate market access for innovative products” [9].

In contrast, India doesn’t have a formalized national HTA process and the public financing of new technologies is very limited [10]. Whereas 60-80% of health care is delivered in the private sector, only 3-5% of the population has health insurance [11] so coverage decisions by insurers have negligible impact on the market uptake. Further, medical practitioners in the private sector are not obliged to follow any official evidence-based guidelines, and continuous medical education is not mandatory [12-14]. Finally, the regulation of medical devices is minimal: in the case of in-vitro tests, only those for HIV, hepatitis B and C and blood typing are considered 'critical’ by the Indian regulator and only these tests must be clinically validated before receiving a license. In this context, our study aimed to provide qualitative insights into the frugal innovation experience of companies that function in an environment that doesn’t have a tradition of indigenous novel bio-medical product development.

Here we present case studies of six private companies in Bangalore, India, that have developed and launched (four cases) or are expecting to soon launch (two cases) devices for the Indian market. These firms belong to a new wave of intellectual property (IP)-based product ventures in the country. We study (i) the evolution of the firms and their approaches to product development; (ii) their funding and human resource challenges; (iii) their access to global science and technology (S&T); (iv) their use of global regulatory requirements, and finally (v) the market challenges that must be overcome in order to access patients with their products. We believe that insights from this study will be of interest to many young companies, regulators and policy makers in the world.

## Methods

We adopted a qualitative case study research methodology that has been used by others to study medical innovation in developing countries [8,15-17]. The innovative medical device industry in India is only emerging today and a quantitative study would not be feasible with such a small sample size. Further, as explained below, the firms have gone through very different paths since their inception and the case study methodology is better suited to capture this heterogeneity. The study protocol was approved by the Ethics Committee of IBAB. Written informed consent was obtained from participants by asking them to positively reply to an interview invitation e-mail. Initially more than 40 private and government doctors, diagnostic labs, manufacturers and distributors of diagnostic tests, NGOs and academics were interviewed about the diagnostics’ business in India. The interviews concerned local innovation versus imported products, delivery of devices to patients in public and government sectors, regulatory issues and doctors’ prescription behaviour. Informants were chosen by purposeful sampling and were chiefly located in metropolitan cities although their experience extended to rural areas as well. Results of these interviews are not presented here, but served to select the six companies located in Bangalore that were the basis for this study. Additionally, the following sources were used: the BioSpectrum India Life Sciences Resource Guide 2010 which is one of the most comprehensive repositories of information on the Indian life science industry (http://www.newindigo.eu/biotech/main/index.htm) and a published review of the Indian biotech industry [18]. Since none of the firms had achieved significant sales at the time of our research, financial measures such as profit, volumes or return on investment could not be used as criteria for selection. Nor were details of debt or equity available for the (largely) privately held companies. We selected medical device firms located in Bangalore, arguably the most innovative biomedical hub in India, that were developing innovative, IP-based products for the Indian market, and low-resource settings in particular. Finally, in the one situation where two companies with similar profiles were identified (that is, inception or origin, type of product and development path) the company that was further in the product development process was chosen. The company-specific interviews sought to understand the inception of the firm and the origin of the key personnel; the path of product development and target product profiles; sources of funding; issues related to clinical validation; regulatory approval and market access in private and government settings. Interviews were not recorded but detailed notes were made during and immediately after each interview. The analysis presented here is based on multiple interviews with the founders of five of the companies. In the case of GE Healthcare India (GEH) the informant was the senior product manager who led the development of MAC400 and MACi. Consequently, the perspective of his own R&D centre may not fully reflect the history of General Electric (GE) in India. In each case there was one interview at the company, followed by a few more conversations in person, by phone or by e-mail. Further information was obtained from company material and other published interviews of the founders. After all the interviews, the write up on each of the six companies was verified by the concerned firm. However the final manuscript was not submitted to them for their verification. All interviews were semi-structured and were conducted between March and December 2011, inclusive.

## Results

### The companies and their products

The firms profiled are XCyton Diagnostics (XCyton), Bigtec Labs (Bigtec), GEH, ReaMetrix India (ReaMetrix), Embrace Global (Embrace) and Achira Labs (Achira). The companies were founded from 2 to 18 years ago (Table 1). Interestingly the founders of these six firms came from six of the nine categories of biotech founders in India, identified previously [18]. The earlier study had pointed out a low rate of company formation by local academics, and that is reflected here, where none is a scientist from local academia (Additional file 1). In terms of the companies’ evolution GEH started as a manufacturing support unit of GE Healthcare Worldwide's Indian manufacturing facility and then evolved into an R&D centre. The remaining ventures started as R&D firms and this has remained unchanged (Additional file 2). Most of the firms were able to take their products from concept to clinical validation in two to three years. The exception was Bigtec where the founders operated in a field that was unfamiliar to them, and where – when the product launches later this year – it will have taken 12 years from the firm’s founding.

**Table 1:** The six companies of this study, their year of formation, their low cost products and the timelines for (i) product concept, (ii) year of first patent being filed and (iii) first sales in India

**Company (Year of formation)**	**Low cost products (Years of product concept, first patent filed and first sales)**
Xcyton (1993)	• CheX: ELISA-based in vitro diagnostic (IVD) kit for HIV patented by the company (1993; 1994; 1998). Technology for the remaining CheX kits was in-licensed from academic institutions and in one case from the Indian division of a multinational company (MNC).
	• XCyto Screen series: PCR-based IVD kits for rapid diagnosis of infectious diseases (2003; 2006; 2011). XCyton owns the IP related to most of the tests, although for the kits for eye infections and sepsis it belongs to the Indian Council of Scientific and Industrial Research which co-financed the research. Also, patents are yet to be filed for genotyping the Human Papilloma Virus.
Bigtec (2000)	• Micro-PCR device and reagents for low-throughput, rapid, point-of-care in vitro diagnosis of infectious diseases, suitable for harsh conditions (2002; 2006; 2012 expected)
GEH (2000)	• MACi: Portable ECG machine (2008; 2008; 2010)
ReaMetrix (2003)	• Dry-Tri: Cold-chain independent and easy to use CD4/CD8 assay reagent for HIV management, suitable for harsh conditions (2004; no patent filed; 2007).
	• Fluorescence reader for CD4/CD8 assay (2007; 2011; 2011).
Embrace (2008)	• Portable and safe infant warmer that helps to preserve mother-child bonding and can work for 4 hours without electricity. (2007; 2008; 2011)
Achira (2009)	• Immunoassay-based microfluidic chips and point-of-care device for low-throughput rapid in vitro diagnosis (biochemistry), suitable for low-resource settings (2009; 2009; external validation planned)
	• Immunoassay-based fabric chips for device-free, point-of-care in vitro diagnosis of infectious diseases (2010; 2010; internal optimisation ongoing)

Each firm wanted its products to be appropriate for use in low-resource settings which constitute the bulk of the Indian market both in volume and in overall value. Thus, each company undertook an independent assessment of the needs of health-care providers in such settings. It went on to construct product profiles according to its own market- and consumer-research without guidance from national health-care payers or regulators. Low-cost was therefore a common criterion, although other specifications varied with the company (Additional file 3). Each firm’s route to its product(s) is outlined below.

a. XCyton develops diagnostic kits for infectious diseases. It has relied on (i) an invention sourced from the local R&D centre of a multinational company (MNC) (one case), or (ii) science sourced from or products developed in collaboration with Indian public or private research institutions (11 cases). These scientific collaborations were enabled by the personal contacts and informal links of the founder to local scientists. They were unofficial collaborations with low administrative burden and great flexibility in negotiation. Initially, XCyton developed ELISA-based kits (CheX) which require a generic reader but are relatively easy to perform even by untrained manpower. Later, the company developed polymerase chain reaction (PCR)-based kits (XCyto Screen) which call for skilled staff and dedicated laboratory facilities. This shift from rapid kits to high-resource technology was partially motivated by fading confidence in the public health-care market.

b. ReaMetrix started out as a contract research organization (CRO) offering services to Western clients. This led to a gradual build up of its capabilities and capacity. Subsequently the firm changed track and developed a proprietary dried reagent tailored to the needs of the National AIDS Control Organization (NACO) program which covers approximately 50% of the patients on anti-retroviral treatment in India. This reagent is used for a flow-cytometer-based test which monitors the patient’s absolute CD4+ and CD8+ T-cell counts and can replace a more expensive product supplied to NACO by an MNC. Also, it (i) removes the necessity of both cold-chain distribution (storage and transport) and on-bench refrigeration and (ii) reduces the possibility of procedural errors by supplying the pre-weighed reagent in ready-to-use disposable tubes. The company went on to develop a cheaper, simpler and more robust fluorescence reader that can replace the flow-cytometer that was supplied to NACO by the MNC. The company estimates that the currently used instrument costs $20,000–90,000 and it is willing to offer its reader at $15,000–20,000. In resource-limited settings it would offer a reagent rental scheme wherein the cost of ownership of the machine is zero. However, disappointed with the government market, the company is considering re-inventing itself yet again to build advanced R&D instruments for Western markets.

c. Initially Bigtec worked on a recombinant insulin for the Indian market. Subsequently it shifted to an innovative PCR-based microfluidics platform for the detection of infectious diseases specific to India. Notably, the founders were not microfluidics’ specialists. They were nevertheless attracted to this technology because it offers the automation and short sample processing times necessary in point-of-care settings. The diagnostic device allows sample preparation and mixing, bio-chemical reactions and sample screening and detection to be performed on a single chip. The diagnosis takes 45 minutes rather than several hours, and can be performed in harsh environmental conditions by an untrained person. The technology has been clinically validated for several diseases. Bigtec is planning to price the device below the cost of a real-time PCR machine. The cost of running a test would be similar to that with a currently available in-vitro diagnostic (IVD) kit for the concerned infection.

d. GEH started as a low cost, off-shored manufacturing unit of the mother MNC. Subsequently, it developed the MAC400 electrocardiogram (ECG) device for emerging markets by removing some features from an existing GE model. It was the first product released for the Brazil, Russia, India and China (BRIC) markets and was priced at $800, compared with GE’s other hospital-class ECG units that had a price tag between $2,000 and $10,000. However, the development of the next ECG device, MACi, was specific to the Indian market. The needs of rural health-care practitioners were surveyed by engineers from several Indian states. This was felt to be a necessity in a country with a multitude of local languages, a range of geographies and wide disparities in income-levels. It featured a fast-charging, long-life battery and was robust and portable. Also, the company realized that the poorly-regulated local market was dominated by very low-cost ECG machines, which was rather unique among BRIC countries. MACi was therefore priced at $500. It was released on the market just one year after product conceptualization. The emergence of GEH as an innovative product development centre capable of the entire design, development and manufacturing of a product was enabled by two key factors: extensive supervision and deliberate technology transfer from GE R&D units located in Germany and the US, as well as the initiative and corporate advocacy of a team at GEH for the development of a product tailored to the Indian market.

e. Embrace was set up to develop and commercialize a portable and safe warmer for low-birth infants. Although initially based in the US, it relocated to India, where the core R&D team made field trips to rural and urban settings in order to consult with potential end-users. One version of the warmer has been developed for use in hospitals and clinics. In the former setting it facilitates the inter-ward transfers of infants which might take up to 40 minutes. It is available for less than $300 compared to $580–$1900 for currently used radiant warmers. Another version is being developed for use at home and in rural settings. In all settings, Embrace’s warmers would replace potentially dangerous electric radiators which can accidentally catch fire.

f. Achira was set-up to capitalize on the founder’s academic expertise in microfluidics. Although it started out intending to provide such services to large global pharmaceutical companies, it soon shifted focus to developing a lab-on-chip platform for low-resource health-care providers in India. Achira is developing two immunoassay-based platforms: (i) microfluidic chips with a dedicated fluorescence reader for quantitative assays and (ii) device-free silk fibre-based chips for qualitative assays, which can be read by the naked eye. The former technology generates results in less than 30 minutes and can be used with minimal technical training. It has been internally validated by the company and external validation is planned. The latter technology is currently being optimized. It is superior to the currently available lateral-flow technology because multiple types of tests can be performed on a single chip. Its large-scale manufacture requires only low-cost physical infrastructure and therefore it will be sold at a lower price than the first platform.

In order to protect their inventions, all the firms filed patents, in India and in other countries. There was a general tendency to first file the applications in India and then in the US and Europe. However, we formed the impression that the young companies did not have an established IP policy.

### Human resources

The Indian medical industry has traditionally been based on reverse-engineering, and therefore many skills required for the development of entirely novel products are rare in the country today [19]. Consequently the companies faced a few challenges related to the recruitment and retention of appropriately skilled personnel. (i) Indians returning from Western nations, with postgraduate academic degrees or industry experience, played an important role in most of the firms (Additional file 1). The process has been accelerated by both the recent economic growth in urban India and the economic stagnation of Western economies. (ii) All the firms have found that neither candidates with experience in the local pharmaceutical industry nor graduates of local academic institutions have the right skill sets to work on the design and marketing of innovative products. Whereas senior scientific staff in the companies are able to train new employees in technical skills, finding experienced candidates for market access activities has been a key challenge. (iii) XCyton and Achira, which employ Indian biologists with postgraduate experience, have found that there are cultural issues related to retaining their staff for long periods. As elsewhere, many biologists in India are women, and there is high attrition due to the relocation of those who follow their spouses to other cities. This churn has serious costs for young firms, in terms of both time and money.

### Funding of the companies

The studied companies managed to engage with both local and international investors to fund their R&D programs, without having to forgo majority equity. It turns out that half of the firms were primarily funded from Indian sources and the other half from foreign ones, as detailed below (more details in Additional file 4).

#### Primarily Indian sources

Among the indigenous start-ups, XCyton and Bigtec benefitted from soft loans and small grants for young R&D firms from the Government of India, and this funding was vital. Both companies have had difficulty finding investors who would be willing to fund marketing and distribution activities without taking a majority share. They perceive such offers as unfair since their products have already been clinically validated and therefore the investment would carry relatively low risk. However, XCyton has very recently obtained an equity investment from a US-based entity which will be used mainly for marketing its XCyto Screen services and also to establish new laboratories across the country. Interestingly, this forced the company to discontinue the two approved CheX tests (for HIV and hepatitis C). This was necessary to avoid being classified as a pharmaceutical company under Indian law and it enabled XCyton to finalize the foreign investment deal without government pre-approval.

#### Primarily Western sources

In contrast to the cases above, ReaMetrix was almost entirely dependent on the private money of the founder who has been a serial entrepreneur in the US, and on international private investors. Although GEH is a division of a global corporation, funds for the development of an ECG for the local market were not granted automatically. After the India-based team of engineers took the initiative, their ideas received financial support first from global headquarters and later from a locally created budget. Finally, Embrace was established as a social enterprise and was funded by US-based donors. The founders are now planning to split the enterprise into a non-profit and a for-profit entity, the latter in order to secure the substantial international investment necessary to enable large-scale manufacturing and global marketing.

Overall, the firms’ major struggle was in raising substantial funds for marketing as well as scaling-up manufacture. Apart from Bigtec which formed a product marketing joint-venture with a major Indian diagnostics manufacturer, the companies needed to rely on foreign investment to finance such activities. Some of the firms fear that an investment by an MNC would result in a loss of control of the pricing strategy, and force them to price their products higher than they would wish even in low-resource settings.

### Globalization of science and technology

Overall, the companies value being located in India. It has allowed them to organize frequent field surveys, construct meaningful product specifications and experiment with market access strategies, all with respect to low resource settings. Notably, however, each firm's ability to develop such appropriate technologies was enabled by the founders’ or other key persons’ experience in Western academia or industry (Table 2 and Additional file 1). Contact with global S&T occurred in the local divisions of MNCs (XCyton and GEH) or through returning Indians (the other firms). Also, for some of the firms, pre-existing international links were instrumental in accessing Western clients and/or funding sources, which were essential in the early days (Table 2). Since the availability of manufacturers and suppliers of advanced services and components in India is limited, this posed a challenge to several of the companies. Being a division of an MNC, GEH has an international network of accredited providers which facilitated sourcing of specific components. However other companies had to establish partnerships with industry located in Europe or the US. These were often initiated during global charity or industry meetings or through international academic collaborations (Additional file 5). Thus, medical technology innovation in a developing country can require outsourcing to the West due to the lack of local facilities or expertise.

**Table 2:** The skills acquired or resources accessed due to founders’ or key persons’ prior links to Western industry or academia

**Company**	**Skills**			**Resources**		
	**Science/technology**	**Product development**	**Entrepreneurship**	**Funding**	**Access to clients**	**Access to suppliers**
XCyton	√	√				
Bigtec		√			√ (for its sister IT company)	
GEH	√	√	Not applicable	√ (division of an MNC)		√
ReaMetrix	√	√	√	√	√ (in its CRO period)	
Embrace	√	√		√ (donations)		√
Achira	√					

### Experience with international regulatory authorities

The companies’ international reach concerns not only S&T but also regulatory approval for their products. This is mainly because the regulation of medical devices in India is rudimentary. Further, the regulatory body is not accustomed to licensing innovative products that have not been approved in a developed country. Some firms were dissatisfied with the limited regulation in the country primarily for two reasons: (a) the lack of dialogue and guidance on what specifications a product should meet and (b) unfair competition from manufacturers offering sub-standard and cheaper versions of their innovative products.

In the absence of local regulation, the companies pursued WHO pre-qualification, US Food and Drug Administration (FDA) approval or the CE mark (Additional file 4). It was considered necessary to engage with foreign regulatory agencies not only for their guidance and to distinguish the companies’ innovative products from substandard ones, but also for accessing global markets, including those of low-income countries. Surprisingly, as exemplified by the struggle of XCyton, even WHO pre-qualification involves mobilizing significant resources. Thus, whereas the company’s HIV CheX test was compliant with WHO guidelines, pre-qualification came only after a two-year effort to attract the attention of the relevant officer who was based in Geneva. Notably, this contract gave XCyton global visibility. Subsequently, international organizations have helped the firm obtain accreditation abroad for other tests.

The firms have also pursued international certifications due to the high uncertainty related to the Indian public market, that is discussed further below. The companies that have tried to sell to the Indian government have failed to do so. Therefore, the companies have accessed, or have considered accessing, the local market via funding from foreign donor organizations. For this, international accreditation of their products would be required.

### Accessing the market

Health-care providers in India range from high-end private hospitals manned by highly qualified personnel and equipped with the latest technologies, to public and private rural health-care centres lacking trained staff and with serious shortcomings in basic facilities such as the availability of uninterrupted power and water. This has large implications for the product planning process since there is significant uncertainty regarding the kind of end users and their sample throughput needs, as well as the target price range. The companies’ perception is that whereas high-end settings require high throughput capacity of an instrument and national or international accreditation, other settings primarily require (i) low capital investment and maintenance costs, (ii) low costs to the patient, (iii) resistance to adverse operating conditions and (iv) the equipment should be explicitly designed to facilitate task shifting to lower cadres of workers. Consequently, the companies have found that reaching such complex markets requires more time than product R&D. Notably, the experience of GEH in marketing to high-resource settings in India proved insufficient to access low-resource settings with the MACi. Thus, for the Indian market, the key obstacles to reaching the customer have been: (a) an underfunded and non-transparent government health-care market and (b) a highly fragmented and poorly regulated for-profit private market. In the case of diagnostics, soaring competition among diagnostic labs has increased the occurrence of referral fees that are paid to doctors on a per patient basis. It is also complex and costly to access African countries, even via the WHO purchasing process. XCyton and Embrace said that the largest funding rounds in their existence would be used in large part for marketing and distribution. These obstacles are discussed in Additional file 6.

## Discussion

Five of the six companies discussed here took their products from concept to validation in two to three years. This compares well to the average product lifecycle of 18–24 months estimated by Eucomed, the medical technology industry body in Europe (http://www.eucomed.org). However, the availability of both funding and the human resources necessary to access the market with finished products has been one of the major impediments to the companies. Whereas advanced technological knowledge could be accessed via links to global academic and industry communities, the lack of local regulatory guidance posed a major challenge for product development. Although FDA, CE and WHO certifications are an alternative, the interviewed companies assert that the high cost of such procedures and/or distant location of these agencies are serious obstacles and result in delays. Whereas there is scarcity of literature on the innovative health care industry in India, some of these issues have been reported previously [17,20]. Further, the paucity of institutional health care payers, the fragmentation of private health-care providers and the lack of national consensus guidelines meant that the companies had to use their own resources to educate the doctors and laboratories about their technologies. Notably, the for-profit nature of the private sector demands that when pricing their products, firms must consider both the affordability for the patient and the provider’s desire to generate profits from the provision of a technology [12,21-23]. This market complexity implies that the commercial success and survival of such companies will depend on their ability to develop ground-breaking strategies in the post-R&D phase also.

## Conclusions

We believe that the future of India’s innovative biomedical industry will depend on the upgradation of several national policies. Whereas this study was not designed to inform such policies, and tools such as stakeholder analysis are better suited for this purpose than the case study method adopted here, we would like to make three recommendations for the development of an innovative medical device sector in India: First, the national regulatory bodies need to offer guidance to industry about product development as the FDA, European Medicines Agency and WHO do. Currently, the scientific capabilities of the relevant agencies are inadequate to do this. Second, government procurement of innovative devices needs to be increased. Also, the process to do so should be made more transparent through the incorporation of explicit evidence-based decision making. The UK’s National Institute for Health and Clinical Excellence and similar government agencies in many other European and some Asian countries, such as Japan, Singapore and Malaysia, appraise medical technologies and advise on their financing from public sources. Third, the private healthcare sector requires more regulation. This implies tackling the issue of referral fees and the production of national guidelines for diagnosis and treatment. In many countries that have nationalised health systems this is achieved through close collaboration between the HTA bodies, medical councils that control doctors’ practice and the national health funds or insurers that directly employ most health care professionals. However, it remains to be seen whether such centralized control can or should be achieved in a large and diverse country such as India, that has a health care sector that is highly fragmented and largely private.

## Abbreviations

Achira: Achira labs; Bigtec: Bigtec labs; BRIC: Brazil Russia, India and China; CRO: Contract research organization; ECG: Electrocardiogram device; Embrace: Embrace global; GE: General electric; GEH: GE Healthcare India; HTA: Health technology assessment; IVD: In-vitro diagnostic; MNC: Multinational company; NACO: National AIDS Control Organization; NGO: Non-governmental organization; PCR: Polymerase chain reaction; ReaMetrix: ReaMetrix India; S&T: Science and technology; XCyton: XCyton Diagnostics; IP: Intellectual property; R&D: Research and development.

## Competing interests

GS’s research is funded by Institut Merieux (IM) which has financial interests in the medical technology industry. However IM did not play any role in designing this study, or in any other aspect related to it other than general funding, as indicated below.

## Authors’ contributions

GS proposed the study. SJ performed and analysed the interviews. SJ and GS wrote the manuscript. Both authors read and approved the final manuscript.

## Pre-publication history

The pre-publication history for this paper can be accessed here:

http://www.biomedcentral.com/1472-6963/13/199/prepub

## Supplementary Material

Additional file 1 Detailed profiles and origins of the founders and key people in the studied companies. Click here for file

Additional file 2 Key events in the evolution of each company and sources of innovation. Click here for file

Additional file 3 Key criteria considered by the companies when constructing product profiles for their devices and other issues of product development. Click here for file

Additional file 4 Sources of funding for the studied companies. Click here for file

Additional file 5 Details of the six companies’ pursuit of global (i) science and technology and (ii) regulatory requirements. Click here for file

Additional file 6 Challenges faced by each company in accessing the Government and private markets in India or other developing countries. Click here for file

## References

[B1] Anonymous Medical technology industry in India: riding the growth curve 2010 Deloitte

[B2] WHO Medical devices: managing the mismatch. An outcome of the Priority Medical Devices project 2011 World Health Organization

[B3] Sahayog JS Impressions from a rural laboratory MFC Bull 2006 316 317 April-June:1–4

[B4] Anonymous Incentives and innovative financing for global health product development 2010 The Global Health Technologies Coalition

[B5] Anonymous Innovative financing mechanisms for global health: overview and considerations for U.S. government participation 2011 The Henry J. Kaiser Family Foundation

[B6] Frost L Reich M ACCESS: how do good health technologies get to poor people in poor countries? 2008 Cambridge, Massachusetts: Harvard Center for Population and Development Studies

[B7] Moran M Henderson K Ropars AL McDonald A McSherry L Wu L Illmer A Sturm T Zmudzki F G-FINDER. Neglected disease research and development: new times, new trends 2009 Sydney, Australia: The George Institute for International Health

[B8] Frew SE Kettler HE Singer PA The Indian and Chinese health biotechnology industries: potential champions of global health? Health Aff (Millwood) 2008 27 1029 1041 10.1377/hlthaff.27.4.1029 18607038

[B9] Henshall C Mardhani-Bayne L Fronsdal KB Klemp M Interactions between health technology assessment, coverage, and regulatory processes: emerging issues, goals, and opportunities Int J Technol Assess Health Care 2011 27 253 260 10.1017/S0266462311000262 21756413

[B10] Thatte U Hussain S de Rosas-Valera M Malik MA Evidence-based decision on medical technologies in Asia Pacific: experiences from India, Malaysia, Philippines, and Pakistan Value Health 2009 12 Suppl 3 S18 S25 20586975 10.1111/j.1524-4733.2009.00622.x

[B11] Anonymous Eleventh Five Year Plan 2007–12 2008 Planning Commission, Government of India

[B12] Nandraj S Beyond the law and the Lord: Quality of private health care Econ Pol Wkly 1994 29 1680 1685

[B13] Das J Hammer J Money for nothing: The dire straits of medical practice in Delhi, India J Development Economics 2007 83 1 36 10.1016/j.jdeveco.2006.05.004

[B14] Das J Hammer J Leonard K The quality of medical advice in low-income countries J Econ Perspect 2008 22 93 114 19768841 10.1257/jep.22.2.93

[B15] Kamunyori S Al-Bader S Sewankambo N Singer PA Daar AS Science-based health innovation in Uganda: creative strategies for applying research to development BMC Int Health Hum Rights 2010 10 Suppl 1 S5 21144076 10.1186/1472-698X-10-S1-S5 PMC3001613

[B16] Chakma J Masum H Perampaladas K Heys J Singer PA Case study: India’s billion dollar biotech Nat Biotechnol 2010 28 783 10.1038/nbt0810-783 20697399

[B17] Frew SE Rezaie R Sammut SM Ray M Daar AS Singer PA India’s health biotech sector at a crossroads Nat Biotechnol 2007 25 403 417 10.1038/nbt0407-403 17420744

[B18] Saberwal G New pharma-biotech company formation in India Nat Biotechnol 2006 24 499 501 16680124 10.1038/nbt0506-499b

[B19] Saberwal G Seeding a skilled workforce Nat Biotechnol 2009 27 773 775 10.1038/nbt0809-773 19668184

[B20] Engel N Kenneth J Pai M TB diagnostics in India: creating an ecosystem for innovation Expert Rev Mol Diagn 12 21 24 22133116 10.1586/erm.11.80

[B21] Jarosławski S Pai M Why are inaccurate tuberculosis serological tests widely used in the Indian private healthcare sector? A root-cause analysis J Epidemiol Global Health 2012 2 39 50 Available online 31 January 2012 10.1016/j.jegh.2011.12.001 PMC7320362 23856397

[B22] Bajaj R It is time to wash the linen The Natl Med J India 2007 20 3 147 149 17867621

[B23] Rajagopalan A Misuse of diagnostic tests Indian J Med Ethics 2008 5 121 122 18754235 10.20529/IJME.2008.044

