# Supplementary material for: Case studies of innovative medical device companies from India: barriers and enablers to development
Source: BMC Health Serv Res. 2013 May 30;13:199. doi: 10.1186/1472-6963-13-199 (PMC3669049; doi:10.1186/1472-6963-13-199)
Supplement: Additional file 1 — Detailed profiles and origins of the founders and key people in the studied companies. [file 1472-6963-13-199-S1.doc]

| **Additional file 1. Detailed profiles and origins of the founders and key people in the studied companies.** | | |  |
| --- | --- | --- | --- |
| **Company** | **Profiles** | | **Summary of professional origin** |
| XCyton | Founder(s) | - Dr. Ravi Kumar obtained an MBBS from Jawaharlal Institute of Post-Graduate Medical Education and Research, Puducherry and a PhD in neurochemistry from the Indian Institute of Science (IISc), Bangalore. Later he was a project coordinator and research leader in Astra Research Centre (now AstraZeneca), Bangalore. | - Professional from a local division of an MNC |
|  | Other key people | - Dr. Latha Lakshman has a PhD in human genetics from National Institute of Mental Health and Neuro Sciences (NIMHANS), Bangalore | - Local scientist |
| Bigtec | Founder(s) | - Chandrasekhar Nair is a chemical engineer. Subsequently, he worked in the field of bioprocess modelling in India and Canada. - G. M. Kini holds an MBA from the University of Pune. Later work experience was with Digital Equipment, Siemens, Ernst & Young Consulting India and PricewaterhouseCoopers India. - Sampathgiri has a Bachelors degree in electronics engineering and has experience in software development. Past work experience has been with IBM, Rational, Digital Equipment and Tata Consultancy Services. - Javagal Guru Dutt is an electronics engineer. Work experience has been with Advanced Micronic Devices, Wipro, Cranes Software and CALYX Software. | - Returning Indian - Local divisions of MNCs and local industry |
|  | Other key people | - Dr. B. K. Iyer is a practicing physician specializing in infectious diseases with advisory experience to the pharmaceutical industry in India and abroad. - Farooq Quadri is a specialist in precision manufacturing and quality systems. - A few post-graduate scientific staff | - Professional from local and foreign industry - Returning Indians |
| GEH | Founder(s) | - Oswin Varghese handled the first project for emerging markets (MAC400) and later led the MACi program. Before this he had industry experience in India as a senior engineer. | - Professional from local industry |
|  | Other key people | - Mentoring and supervision by the global GE Healthcare teams (in Germany and the US) who had worked on GE's earlier ECG products. | - Professionals from German and US divisions of the mother MNC |
| ReaMetrix | Founder(s) | - Dr. Bala S Manian is a Silicon Valley serial entrepreneur of Indian origin with a PhD in electronic engineering. He founded several successful companies and has extensive industry expertise in the biological applications of optometrics. ReaMetrix is his first Indian firm. | - Entrepreneur of Indian origin located in the US |
|  | Other key people | - Dr. Steve T. Kunitake is a US scientist with extensive industry expertise in optomechanical systems and biochemistry and assay development. - Other key scientific staff | - Professional from US industry - Returning Indians |
| Embrace | Founder(s) | - Rahul Panicker holds a PhD in electrical engineering from Stanford University, and a B. Tech. from IIT Madras, India. He has had industry experience in the US. - Naganand Murty holds an M.S. in management science and engineering from Stanford University. He has experience in biotech/pharma consulting and venture financing in Europe. - Linus Liang graduated with an M.S. in computer science from Stanford University and a BA in computer science from University of California (UC), Berkeley. Earlier, he had started two other technology companies and also has had experience in the IT industry. - Jane Chen holds an MBA from Stanford University and a Masters in Public Administration from Harvard University. She previously worked with non-profit organizations on health-care issues in developing countries. She also spent several years with international charities. | - Returning Indians and foreigners located in India |
|  | Other key people | - Numerous interns from Stanford University | - Foreigners located in India |
| Achira | Founder(s) | - Dr. Dhananjaya Dendukuri is a returning Indian with a PhD in microfluidics from MIT in the US. - Dr. Suri Venkatachalam did a PhD in condensed matter physics from IISc and post-doctoral work in computational neuroscience from UC, San Diego. After returning to India he briefly worked in the local biotech industry. Subsequently, he founded Achira’s mother company Connexios Life Sciences. He also co-founded Group Lifespring which invested in Achira. | - Returning Indian - Professional from local industry with post-graduate education in the US |
