# Supplementary material for: Case studies of innovative medical device companies from India: barriers and enablers to development
Source: BMC Health Serv Res. 2013 May 30;13:199. doi: 10.1186/1472-6963-13-199 (PMC3669049; doi:10.1186/1472-6963-13-199)
Supplement: Additional file 2 — Key events in the evolution of each company and sources of innovation. [file 1472-6963-13-199-S2.doc]

| **Additional file 2. Key events in the evolution of each company and sources of innovation** | |
| --- | --- |
| XCyton | 1993: Product development company with approximately 10 staff developing relatively simple to use protein-based immunodiagnostic assays.  2003: Launched an additional R&D program for PCR-based rapid detection of DNA of pathogens.  Source of innovation: Indian academic institutions throughout its existence (except the neurocysticercosis test): (i) The HIV test was patented by the firm itself; (ii) the patent for neurocysticercosis was licensed from AstraZeneca, Bangalore where the founder had invented it; (iii) the hepatitis C patent was licensed from International Centre for Genetic Engineering and Biotechnology (ICGEB), New Delhi and (iv) for Japanese encephalitis the product was developed in collaboration with NIMHANS, Bangalore, King George Medical College, Lucknow and National Institute of Immunology, New Delhi. Patents will be filed in the future. The PCR-based kits were developed in collaboration with various Indian research institutions, both private and public. |
| Bigtec | 2000: A member of Bigtec Holdings. R&D company developing affordable health-care products for the Indian market with approximately 10 staff. Pipeline included optimization of a recombinant insulin technology (the clone in-licensed from a UK-based firm) and a microfluidics-based platform for PCR (platform conceptualized in 2002). The low-cost insulin manufacturing technology was not commercialized, possibly due to significant competition from established manufacturers of similar products. R&D financed by a revenue stream from IT (SAP) services to Western clients provided by a sister company. This IT firm has grown from 15 staff in 2000 to 125 staff currently.  2011: The PCR device was clinically validated in collaboration with national Indian medical institutions for the in vitro diagnosis of malaria, hepatitis B, dengue, chikungunya and typhoid-causing Salmonella. Validation for hepatitis C and tuberculosis is in progress.  Source of innovation: In-house throughout its existence (apart from the insulin project). |
| GEH | 2000: GEH's Cardiology Diagnostics' division set up to reduce the cost of manufacturing. Knowledge transfer from GE's teams in Germany and the US.  2004: The division was capable of full system development, and a few ECG products were released for global markets.  2005: The division lobbied corporate headquarters in the US for funding a new ECG product line for emerging markets. The program was funded from a global budget of GE Healthcare Worldwide.  2008: GEH started a sub-team of 9 people developing novel products for low-income settings. The goal was to understand the specific needs of doctors working in low-resource rural settings.  2008: GE Healthcare Worldwide launched the 'In Country For Country' program. In India, it is called In India For India (IIFI) and it is funded by GEH. The IIFI unit has 20 staff and has launched three products, including MACi. MACi didn’t have a dedicated marketing person and its product profile was defined by GEH’s engineering and sales teams.  Source of innovation: In-house throughout its existence. |
| ReaMetrix | 2003: CRO to the life sciences' industry located in the UK and US. The US office of ReaMetrix was instrumental in channeling clients to the Indian branch. The CRO period of the firm was crucial in building in-house expertise and also enabled building high-end facilities.  2004: Product development company focussed on the needs of NACO. It developed an equivalent of an MNC reagent that is better adapted to low-resource settings.  2007: Product development company developing generic and innovative reagents as well as innovative equipment. The device program benefited from the hitherto unutilized expertise of the founder in instrumentation design and opto-metrics.  Source of innovation: In-house throughout its existence. |
| Embrace | 2008: The original product idea crystallized in 2007 as an assignment at Stanford University. The company was incorporated in the US as a social enterprise to participate in a contest organized by Stanford University (Social Entrepreneurship Challenge). This secured seed funding for the firm.  2010: The core R&D team shifted to Bangalore, India, as the founders focused on the Indian market.  Currently, the firm is being split into a non-profit entity focused on low-resource settings and education, and a for-profit entity which will pursue large investment from mostly US-based Venture Capitalists.  2011: Embrace launched sales of one version of the warmer in South India. Other versions are undergoing clinical validation.  Source of innovation: in-house throughout existence; original product concept was born in US academia. |
| Achira | 2008: A division of a Bangalore-based drug-discovery firm Connexios Life Sciences with 5 staff. Initially, it explored the possibility of providing high-value solutions in microfluidics to global pharmaceutical companies.  2009: As Achira’s focus changed to the development of a lab-on-chip platform for low-resource settings, it was spun out from its mother company with 10 staff. Interestingly, this change was suggested by one of the co-founders (who had also co-founded the mother firm) who believed that products relevant to the local market are a better business proposition.  2011: The microfluidic chips with a dedicated fluorescence reader have been internally validated by the company for thyroid and infertility tests and external validation is planned in collaboration with small clinics and hospitals in India. It currently employs 17 staff.  Source of innovation: In-house throughout its existence. Achira’s key patents relate to the method of reagent functionalization inside the chip and a design patent for the fluorescent reader will be filed in the future. |
