# Supplementary material for: Case studies of innovative medical device companies from India: barriers and enablers to development
Source: BMC Health Serv Res. 2013 May 30;13:199. doi: 10.1186/1472-6963-13-199 (PMC3669049; doi:10.1186/1472-6963-13-199)
Supplement: Additional file 3 — Key criteria considered by the companies when constructing product profiles for their devices and other issues of product development. [file 1472-6963-13-199-S3.doc]

| **Additional file 3. Key criteria considered by the companies when constructing product profiles for their devices and other issues of product development.** | | |
| --- | --- | --- |
| **Company** | **Key criteria for product profiles** | **Other aspects of product development** |
|  | **Common to all**   - Focus on low-resource settings - Low-cost |  |
| XCyton | - Mapping of unmet clinical needs in the Indian market - Focus on infectious diseases specific to India and other developing countries | - Availability of suitable academic sources of innovation |
| Bigtec | - Focus on infectious diseases specific to India and other developing countries | - Feasibility of product manufacturing in India - One of the key challenges was that the silicon microchips typically used in micro real-time PCR devices have extensive patent coverage, which would have dramatically increased the cost of a test. The company solved this issue by designing a disposable ceramic microchip which is one of their key IP assets. - Bigtec sources the chips from manufacturers in the EU and the US as micro level machining and engraving foundries do not exist in India. However, the PCR device is entirely manufactured in India. |
| GEH | - Cutting the cost of a device developed for Western markets (MAC 400) - Focus on the cost and specific needs of low-resource settings (MACi): portable, robust design, fast battery charging, longer battery life, advanced interpretation software (first time for a product at that price). Though designed for India, received very good response from European practitioners. - Compliance with global product standards imposed by GE policy | - Availability of cheap components (changing traditional providers to cheaper ones and replacing customized components by off-the shelf ones) - One key invention developed specifically for the Indian market was a dynamic AC current noise filter that handles the frequent voltage fluctuations characteristic of the country’s electric supply system, although this feature is not needed when the machine is operated in battery mode. - As there are no regulations in India for a medical device, local players can introduce devices of lower standard at very low cost, and such players have 70% of market share. This is unique for India, as compared to China or Brazil. - In order to avoid “product cannibalization” among GEH’s range of ECG machines this version did not feature its proprietary automatic ECG interpretation algorithm. However a pilot market release revealed that this feature is essential for rural doctors who are rarely trained as cardiologists, and an algorithm-enabled version of MACi was released the following year without a price increase |
| ReaMetrix | - Extensive mapping of market niches for future products in the Indian market - Focus on diseases specific to India and other developing countries | - Lack of regulatory or clinical guidelines in India: significant internal effort was required to frame the product development process and desired product specifications |
| Embrace | - “Human-centric” product development addressing an unmet need. The design was around two principles: the device should operate for up to four hours without electricity and should be placed close to the mother to preserve the mother-infant bonding. - Replacing unsafe electric radiators. | - Feasibility of product manufacturing in India. In particular, availability of ISO-certified components in India - Manufacture of components is outsourced to off-shore locations. Assembly and quality control are done in India. |
| Achira | - Capitalizing on the founder’s academic expertise in microfluidics. - The microfluidic chips with a dedicated low-throughput fluorescence reader will target the fragmented private health-care market. - Conversely, the silk fabric chips which do not require a fluorescence reader will be cheaper and for the government health market. | - Feasibility of product manufacturing in India. As happened at Bigtec, Achira needed to import many essential components for chip and instrument fabrication. It has now developed a large-scale chip manufacturing process that can be carried out entirely in India. |
