# Supplementary material for: Case studies of innovative medical device companies from India: barriers and enablers to development
Source: BMC Health Serv Res. 2013 May 30;13:199. doi: 10.1186/1472-6963-13-199 (PMC3669049; doi:10.1186/1472-6963-13-199)
Supplement: Additional file 4 — Sources of funding for the studied companies. [file 1472-6963-13-199-S4.doc]

| **Additional file 4. Sources of funding for the studied companies** | | | |
| --- | --- | --- | --- |
| **Company** | **0–2 years** | **3–5 years** | **After 5 years** |
| *Primarily Indian sources* | | | |
| XCyton | - Private angel funding | - Venture capital from the Small Industries Development Bank of India and Canara Bank | - Revenue from sales of diagnostic kits (mostly rapid HIV test CheX) and diagnostic services - Soft Loan from the Government of India's New Millennium Indian Technology Leadership Initiative (NMITLI) - Private Indian (2006) and US (2011) investors |
| Bigtec | - Personal money of the founders - Revenue stream from the sister IT firm | - Revenue stream from the sister IT firm | - Soft loans and small grants from NMITLI, Indian Council of Medical Research and the Department of Biotechnology, Government of India - Private Indian equity investments - Revenue stream from the sister IT firm (the reliance on this funding is decreasing) - A grant from the Grand Challenges Canada, Point-of-Care Diagnostics 2011 |
| Achira | - Angel funding from Group Lifespring, a member of the Nadathur Group (which is also a strategic investor in its mother firm Connexios Life Sciences). - A grant from the Grand Challenges Canada, Point-of-Care Diagnostics 2011 | Not applicable | Not applicable |
| *Primarily Western sources* | | | |
| GEH | - Funded as a division of GE Healthcare Worldwide (as an engineering support team for an off-shored manufacturing unit) | - Dedicated product development funding from GEH | - Funds from the 'In India For India' (IIFI) program of GEH |
| ReaMetrix | - Personal money of the founder from previous entrepreneurial ventures | - Revenue from CRO services to UK- and US-based clients | - Revenue from sales of diagnostic kits - International angel investors |
| Embrace | - Stanford University's 'Social Entrepreneurship Challenge' seed grant - Family and individual donations (US-based) | - International fund-raising planned. | Not applicable |
