# Supplementary material for: Case studies of innovative medical device companies from India: barriers and enablers to development
Source: BMC Health Serv Res. 2013 May 30;13:199. doi: 10.1186/1472-6963-13-199 (PMC3669049; doi:10.1186/1472-6963-13-199)
Supplement: Additional file 5 — Details of the six companies’ pursuit of global (i) science and technology and (ii) regulatory requirements. [file 1472-6963-13-199-S5.doc]

| **Additional file 5. Details of the six companies' pursuit of global (i) science and technology and (ii) regulatory requirements.** | | |
| --- | --- | --- |
| **Company** | **Links to EU- or US-based industry, academia or charities** (excluding prior international connections of the founders or key persons detailed in Supplementary Table 1) | **Compliance with global regulatory requirements and issues arising from the limited regulation in India** |
| XCyton | - JEV CheX technology developed at the Indian captive R&D centre of an MNC - Collaboration with the Bill and Melinda Gates Foundation on endorsement of JEV CheX by WHO | - CheX kits for HIV and hepatitis C comply with WHO specifications - JEV CheX tested by The Health Protection Agency, UK and currently in the process of obtaining the CE mark. If successful, XCyton is willing to partially reimburse the costs of this process to the Agency. - Whereas XCyton is pursuing the Indian National Accreditation Board for Testing and Calibration Laboratories (NABL) voluntary license for its diagnostic lab, only the costly US FDA Clinical Laboratory Improvement Amendments (CLIA) accreditation, which it hopes to obtain, will enable the firm to certify a commercial version of the PCR-based diagnostic products. - XCyton's diagnostic lab has ISO certification. |
| Bigtec | - Sourcing certain device parts from Europe and the US - Collaboration with Western academia - Grantee of the Grand Challenges Canada, Point-of-Care Diagnostics competition | - Internal product development was compliant with CE mark requirements. Bigtec found this helpful for two reasons: it facilitated the drawing up of product specifications and also makes the product ready for global sales. The CE application is ongoing. - Whereas the Indian regulator only requires extensive clinical validation for the hepatitis B test, the company pursues the same internationally accepted standards for all its products. |
| GEH | - Extensive mentoring from GE Healthcare teams in Germany and the US - GE suppliers of components are based in Asia, Europe and the US | - GE corporate policy requires that all products and their components are compliant with global standards (of the EU and US). - However, GEH feels that there is aggressive price competition from local manufacturers of sub-standard devices, which is due to the lack of regulation in the country. |
| ReaMetrix | - In the CRO period, some projects for UK- and US-based clients contributed to building in-house expertise in protein modification and assay development - Scientific inputs from US-based industry - Sourcing of certain device parts and reagents from Europe and the US - Collaboration with the Foundation for Innovative New Diagnostics (FIND) on quality control for rapid malaria tests | - FDA non-inferiority approval for the firm's HIV dried reagent - Key reagents sourced from WHO-accredited suppliers - ReaMetrix saw the lack of local regulatory guidance as impeding its product development process and it is of the opinion that such guidance would be beneficial for the entire biotech industry in India. |
| Embrace | - Key advisory persons are located in the US - Certain prototyping projects outsourced to the US | - Internal product development was compliant with CE mark requirements. Embrace found this helpful for two reasons: it facilitated the drawing up of product specifications and also makes the product ready for sales to international non-profit organizations and to access global markets directly. - In addition to clinical trials in India, completed trails in the US to secure international credibility. - Embrace has ISO certification - Whereas the baby warmer does not have competing products on the Indian market, potentially harmful electric radiators are widely used in the unregulated private and government health care settings. |
| Achira | - Scientific inputs from US-based academia and industry - Sourcing certain device parts from Europe and the US - Grantee of the Grand Challenges Canada, Point-of-Care Diagnostics 2011 competition for the silk fabric-based platform | - Achira feels that it is beneficial to an innovative company that the Indian medical device market is not “overregulated”. This should enable it to clinically validate a pilot version of its product and experiment with small-scale sales faster and cheaper than would be required in the EU or the US. - Once a final product version is defined and validated, Achira plans to apply for the CE mark. |
