# Supplementary material for: Case studies of innovative medical device companies from India: barriers and enablers to development
Source: BMC Health Serv Res. 2013 May 30;13:199. doi: 10.1186/1472-6963-13-199 (PMC3669049; doi:10.1186/1472-6963-13-199)
Supplement: Additional file 6 — Challenges faced by each company in accessing the Government and private markets in India or other developing countries. [file 1472-6963-13-199-S6.doc]

| **Additional file 6. Challenges faced by each company in accessing the Government and private markets in India or other developing countries.** | | |
| --- | --- | --- |
| **Company** | **Government market** | **Private market** |
| XCyton | - Very difficult to sell to the government sector in India - Successful contract with WHO for sales to governments in India, Bangladesh and Sri Lanka (over 2 million HIV tests so far). Even after this, the company did not manage to access the Indian public market directly. - Accessing African countries very difficult since separate interaction with each government is necessary | - Pathology labs of large chains very difficult to access due to competition from local and MNCs' products - Smaller independent private labs and hospitals have been accessed but with diagnostic services, rather than products. - Necessary to focus significant marketing efforts on doctors who prescribe diagnostics. - Soaring competition among diagnostic labs for referring doctors has increased the occurrence of referral fees. Doctors are paid such fees on a per patient basis and they are often a percentage (10–40%) of the price that patients pay the lab. - The recently completed, largest funding round to date will be used for marketing and distribution |
| Bigtec | - Unsure if further product development should target the government market and if so, with what product specifications. | - Recently formed a joint-venture for marketing and distribution with a large Indian firm, Tulip Diagnostics. - Concerned that the majority of patients in India would not be able to buy their products in the private market at a price that would also make the business viable. - Soaring competition among diagnostic labs for referring doctors has increased the occurrence of referral fees. Doctors are paid such fees on a per patient basis and they are often a percentage (10–40%) of the price that patients pay the lab. |
| GEH | - Fragmented sales to some medical colleges and rural health care centres | - Rural private health care market highly fragmented. - Unfair competition from manufacturers of sub-standard ECG devices due to poor market regulation. - Non-compliance by independent distributors led to a decision to employ direct sales team. A project currently being piloted assumes hiring a team of 100 who will reach tier 2 and 3 towns. Private doctors can buy MACi in monthly instalments of about Rs. 800 for 3 years. This should allow the cost of an ECG to come down from the current Rs. 150 or more, to about Rs 10. - As all GEH products must comply with global regulatory requirements, the low-cost ECG machines were also released in the EU, where MACi received a surprisingly good market uptake. |
| ReaMetrix | - Very difficult to sell to the government sector in India. Whereas the reagent obtained a non-inferiority approval from the US FDA versus the Becton Dickinson TriTEST reagent, was superior to its competitor (in terms of not requiring a cold chain) and was less expensive (Rs. 125 versus Rs. 600–750 per test), the company’s attempts over two years to access NACO failed and the national program continues to purchase the original reagent. - Accessing African countries very difficult since separate interaction with each government is necessary. - Accessing national markets via WHO pre-qualification not attempted because of the high amount of paperwork involved. | - Unfair competition from manufacturers of sub-standard diagnostics due to poor market regulation. - Pathology labs of large chains very difficult to access due to competition from MNCs' products. - Smaller independent private labs and hospitals have been accessed. - Necessary to focus significant marketing efforts on doctors who prescribe diagnostics. - Soaring competition among diagnostic labs for referring doctors has increased the occurrence of referral fees. Doctors are paid such fees on a per patient basis and they are often a percentage (10–40%) of the price that patients pay the lab. |
| Embrace | - Preliminary attempts are being made to access the government market but financing for this type of device is particularly scarce. - Unsure if further product development should target the government market and if so, with what product specifications. | - Rural private health care market highly fragmented. - Necessary to focus significant marketing/educational efforts on individual doctors. - Shortage of distributors and sale representatives who can sell an innovative product. Thus, “concept marketing” skills are generally unavailable in India. Marketing strategies in India are largely based on new variations of existing products, whereas the warmer is a unique product in this category and at this price point. Consequently, Embrace employs a direct sales team. A tie-up with an MNC for global, non-exclusive distribution is being considered. - Doctors are interested to rent the device to patients in order to secure continuous revenue. Thus, another version of the warmer is being developed for home use. - Pursuing the largest funding round so far, to be used in large part for marketing and distribution. |
| Achira | - Unsure if further product development should target the government market and if so, with what product specifications. | - Concerned that the majority of patients in India would not be able to buy their products in the private market at a price that would also be viable from the perspective of its business. |
